# Correction: Inhibition of platelet activation suppresses reactive enteric glia and mitigates intestinal barrier dysfunction during sepsis

**DOI:** 10.1186/s10020-022-00587-1

**Published:** 2022-12-09

**Authors:** Bo Cheng, Mengyu Du, Shuxuan He, Lan Yang, Xi Wang, Hui Gao, Haiqing Chang, Wei Gao, Yan Li, Qiang Wang, Yansong Li

**Affiliations:** 1 Department of Anesthesiology & Center for Brain Science, The First Affiliated Hospital of Xi’an Jiaotong University, Xi’an, 710061 Shaanxi China; 2 Department of Anesthesiology, The First Affiliated Hospital of Wenzhou Medical University, Wenzhou, 325000 Zhejiang China

**Correction: Molecular Medicine (2022) 28:127** 10.1186/s10020-022-00556-8

Following publication of the original article (Cheng et al. 2022), the authors informed us that in Figs. 1g and 5g, they mistakenly used the HE image of sham + Cliostazol group and the HE image of sham + 6877002 group. This correction does not affect the results and the conclusions of the study. The correct Figs. 1 and 5 are given in this correction.

**Fig. 1:**
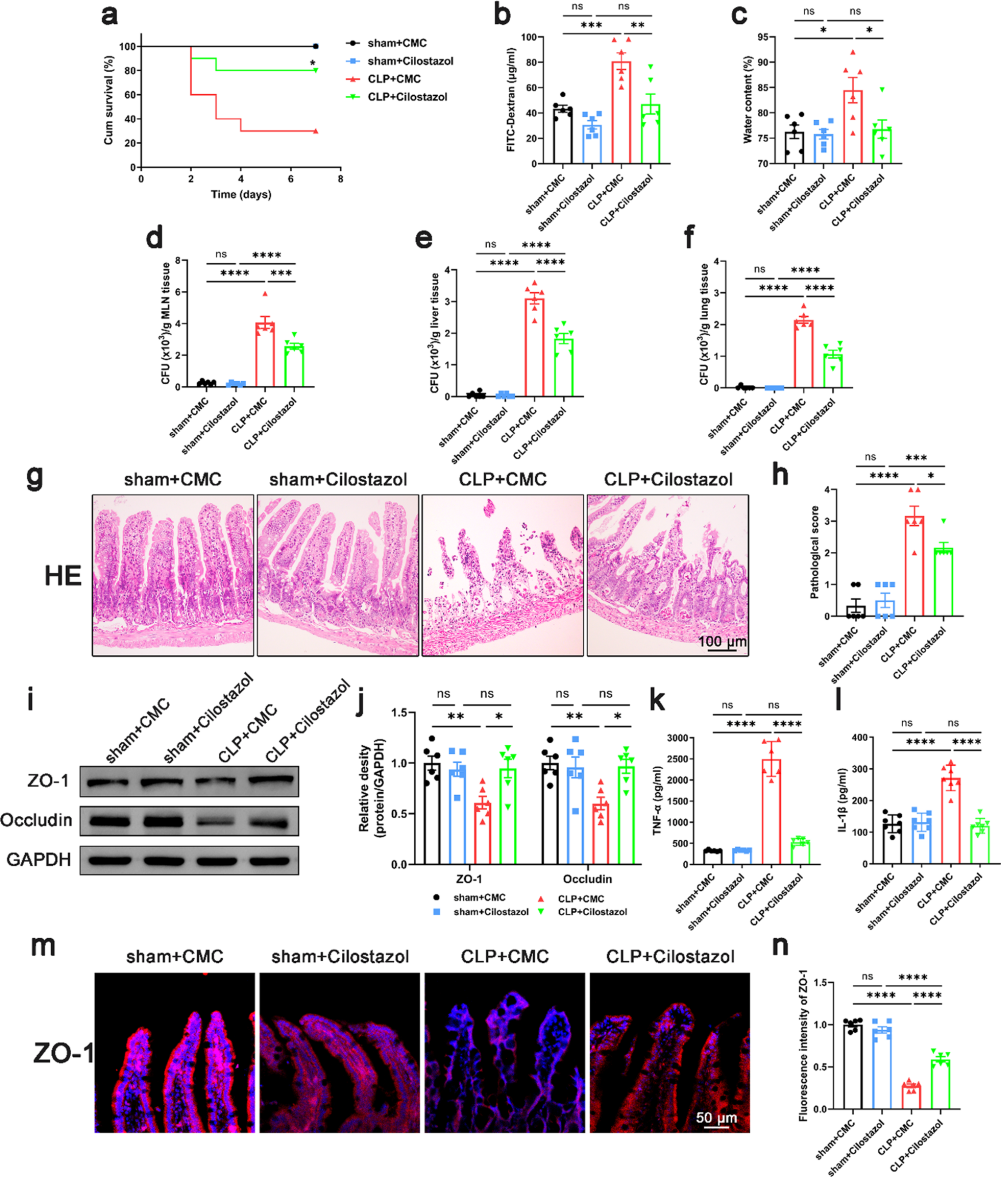
Effect of inhibiting platelet activation on intestinal barrier function in septic mice. Cilostazol (10 mg/kg) was administrated orally 2 h prior to and at 12 h after CLP to inhibit platelet activation. 24 h after CLP, mice were sacrificed, and tissue samples were collected. **a** The survival rate of the mice within 7 days after CLP was observed by survival curves (n = 10). **b**–**f** Intestinal barrier permeability was indicated by serum FITC-Dextran levels (**b**) (n = 6), water content (**c**) of gut (n = 6), and colony-forming units (CFUs) (**d**–**f**) from mesenteric lymph node (MLN), liver and lung (n = 6). **g**, **h** Haematoxylin and eosin (H&E) staining and pathological score, Scale bar = 100 μm (n = 6). **i**, **j** Western blot analysis of ZO-1 and occludin expression (n = 6). **k**, **l** TNF-α and IL-1β levels in intestinal tissues (n = 6). **m**, **n** Immunofluorescence staining analysis of ZO-1 (red), Scale bar = 50 μm (n = 6). The data are presented as the mean ± SEM, *P < 0.05, **P < 0.01, ***P < 0.001, ****P < 0.0001

**Fig. 5:**
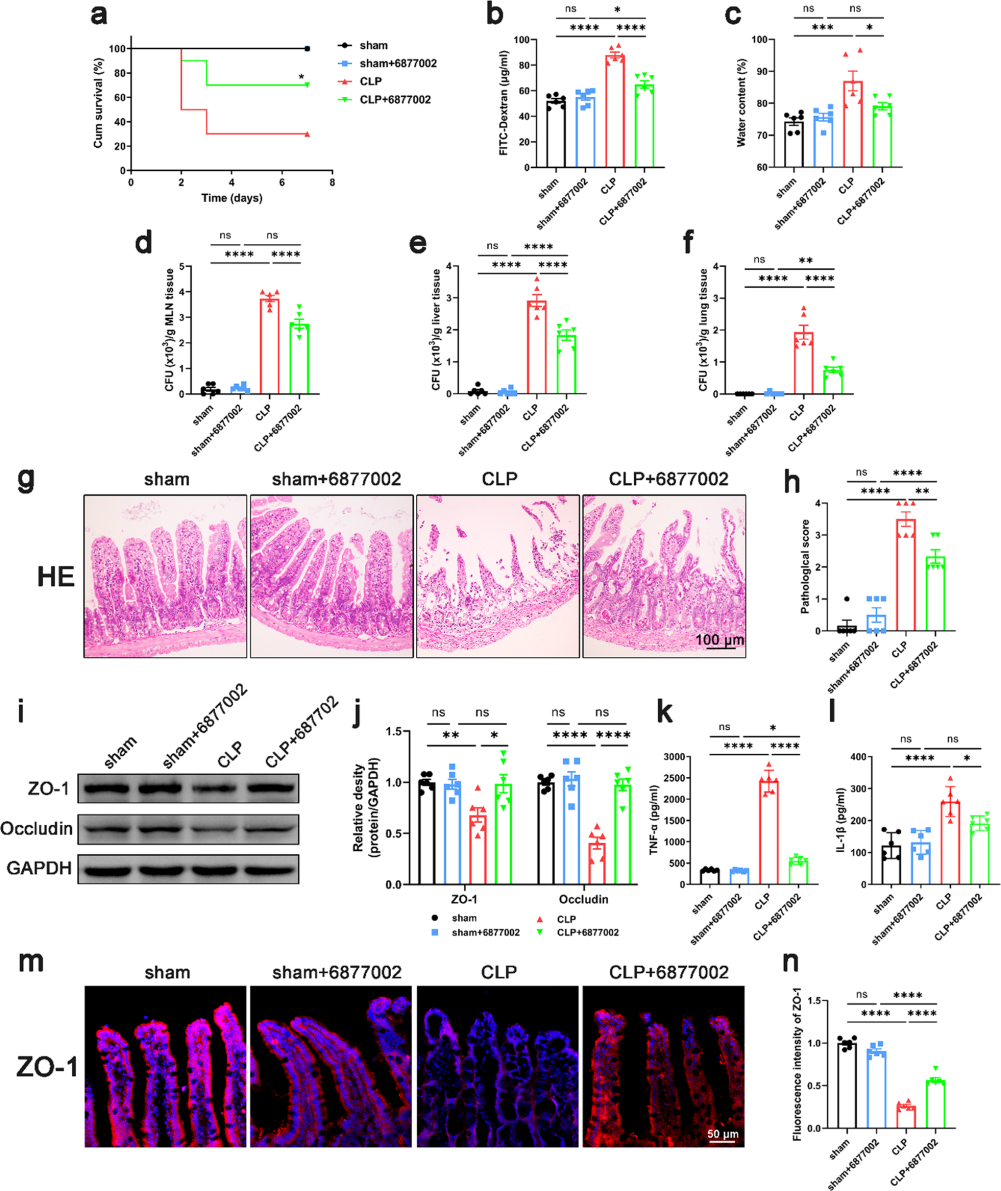
Blocking CD40L–CD40–TRAF6 signaling pathway improved intestinal barrier function in septic mice. Compound 6877002 (10 μmol/kg) was injected intraperitoneally 2 h prior to and at 12 h after CLP to block the CD40–TRAF6 signaling pathway. 24 h after CLP, mice were sacrificed, and tissue samples were collected. **a** The survival percentage of the mice was investigated within 7 days after CLP by survival curves (n = 10). Intestinal barrier permeability was indicated by serum FITC-Dextran levels **b** (n = 6), water content (**c**) of gut (n = 6), and colony-forming units (CFUs) **d**–**f** from mesenteric lymph node (MLN), liver and lung (n = 6). **g**, **h** Haematoxylin and eosin (H&E) staining and pathological score, Scale bar = 100 μm (n = 6). **i**, **j** Western blot analysis of ZO-1 and occludin expression (n = 6). **k**, **l** TNF-α and IL-1β levels in intestinal tissues (n = 6). **m**, **n** Immunofluorescence staining analysis of ZO-1 (red), Scale bar = 50 μm (n = 6). The data are presented as the mean ± SEM, *P < 0.05, **P < 0.01, ***P < 0.001, ****P < 0.0001

Also, an error was identified in the Results section. The updated section is given below:

**Results**

**Blocking CD40L–CD40–TRAF6 signaling pathway improved intestinal barrier function in septic mice**

These results suggested that blocking CD40L–CD40–TRAF6 signaling pathway may be involved in the efficacy of cilostazol in ameliorating intestinal barrier dysfunction.

The original article (Cheng et al. 2022) has been corrected.
