# Supplementary figures and images for: Inhibition of platelet activation suppresses reactive enteric glia and mitigates intestinal barrier dysfunction during sepsis
Source: Mol Med. 2022 Oct 27;28:127. doi: 10.1186/s10020-022-00556-8 (PMC9615156; doi:10.1186/s10020-022-00556-8)

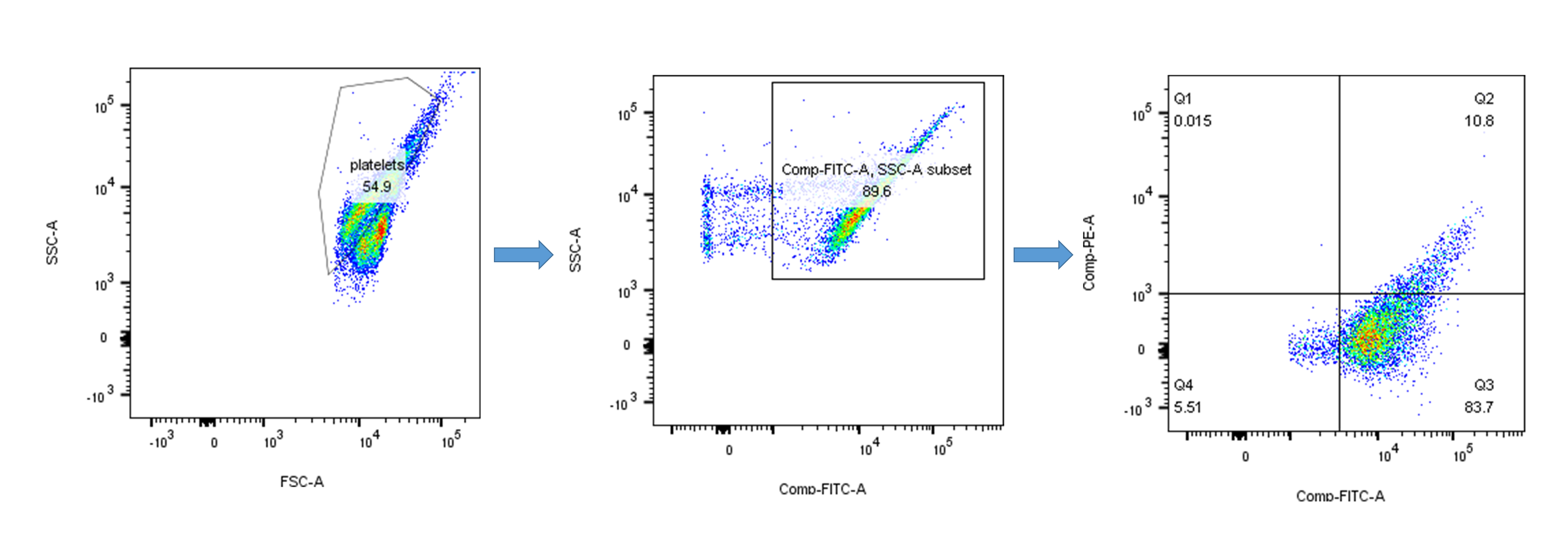

Supplement: Supplementary file 1 — Additional file 1: Fig. S1. The gating strategies for the flow cytometry experiments. Data analysis was performed using FlowJo (Ashland, OR). Scatter and staining with the FITC-anti-CD41 and PE-anti-CD40L antibodies were used to gate platelet population. Cells were first gated by regions within a side scatter area (SSC-A) versus forward scatter area (FSC-A) plot, and then through gating those populations in the SSC-A versus FITC-A plots. Activated platelets were defined as FITC-anti-CD41-A positive and PE-anti-CD40L-A positive (Figure S1). [file 10020_2022_556_MOESM1_ESM.tif]

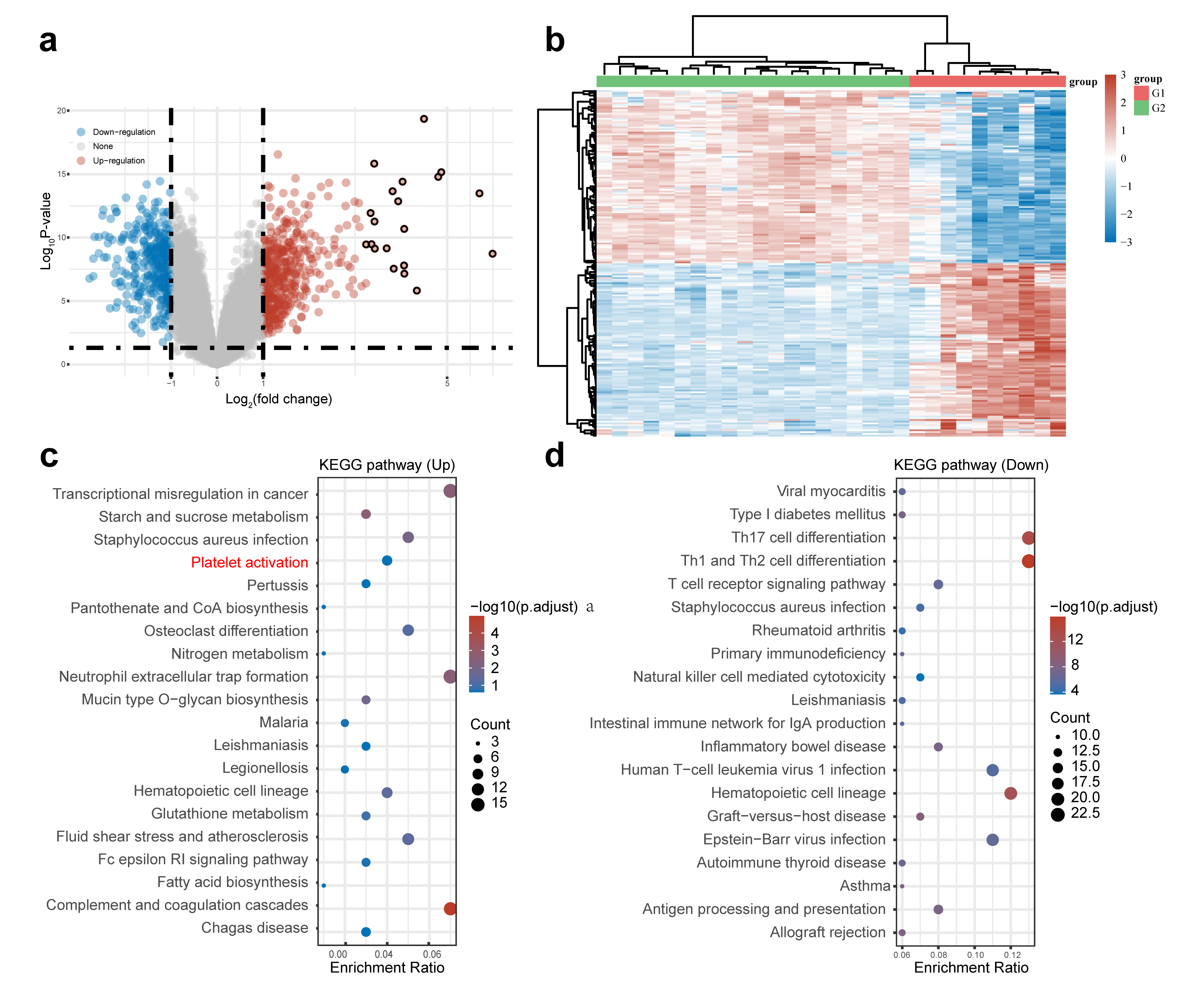

Supplement: Supplementary file 2 — Additional file 2: Fig. S2. KEGG enrichment of differential expressed genes in septic patients compared with healthy person. a The volcano plot was constructed using the fold change values and P-adjust. Red dots indicate upregulated genes; blue dots indicate downregulated genes. b The heatmap of the differential gene expression, where different colors represent trends of gene expression in different tissues. c, d The enriched KEGG signaling pathways were selected to demonstrate the primary biological actions of major potential mRNA. Colors represent the significance of differential enrichment; the size of the circles represents the number of genes. In the enrichment result, P < 0.05 or FDR < 0.05 is considered to be a meaningful pathway (enrichment score with − log10 (P) of more than 1.3). [file 10020_2022_556_MOESM2_ESM.tif]

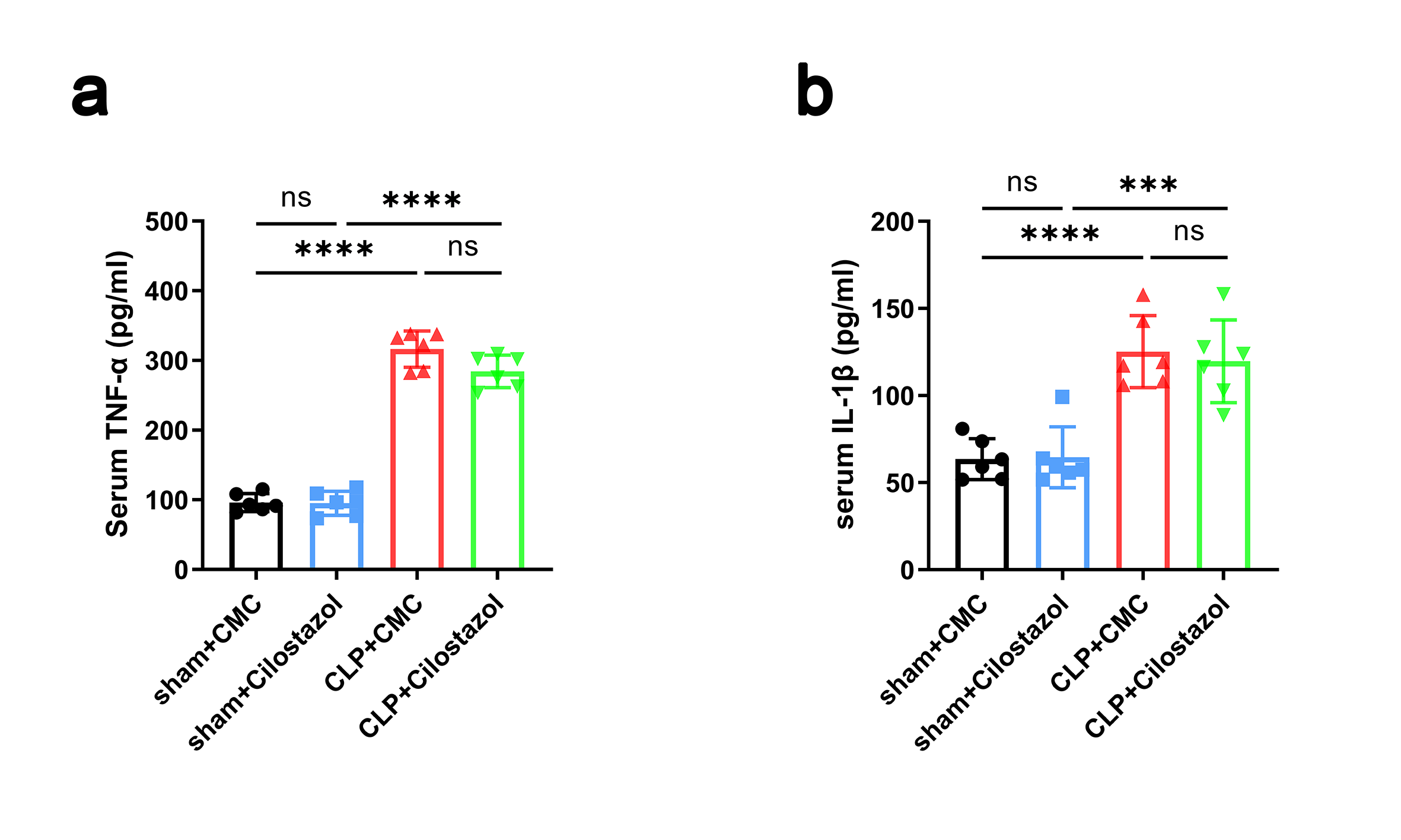

Supplement: Supplementary file 3 — Additional file 3: Fig. S3. Effects of cilostazol treatment on serum inflammatory factors. The level of serum TNF-α (a) and IL-1β (b) in each group (n = 6). The data are presented as the mean ± SEM, ***P < 0.001, ****P < 0.0001, and ns indicates no significant difference. [file 10020_2022_556_MOESM3_ESM.tif]

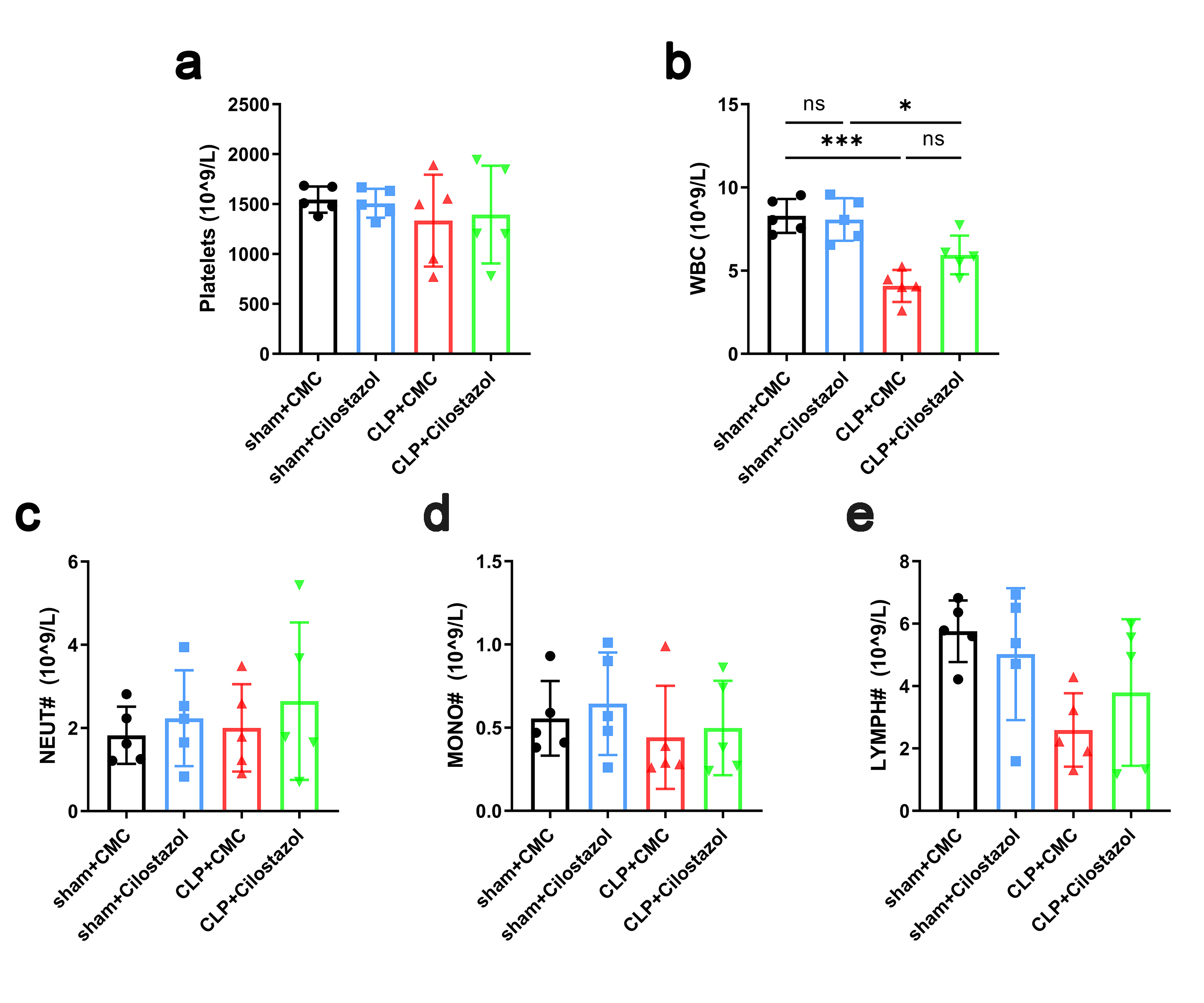

Supplement: Supplementary file 4 — Additional file 4: Fig. S4. Effects of cilostazol treatment on complete blood counts after CLP. a, b The level of platelets (a), WBC (b), NEUT (c), MONO (d) and LYMPH (e) (n = 5). WBC, white blood cells; NEUT, neutrophil; MONO, monocyte; LYMPH, lymphocyte. The data are presented as the mean ± SEM, *P < 0.05, ***P < 0.001, and ns indicates no significant difference. [file 10020_2022_556_MOESM4_ESM.tif]

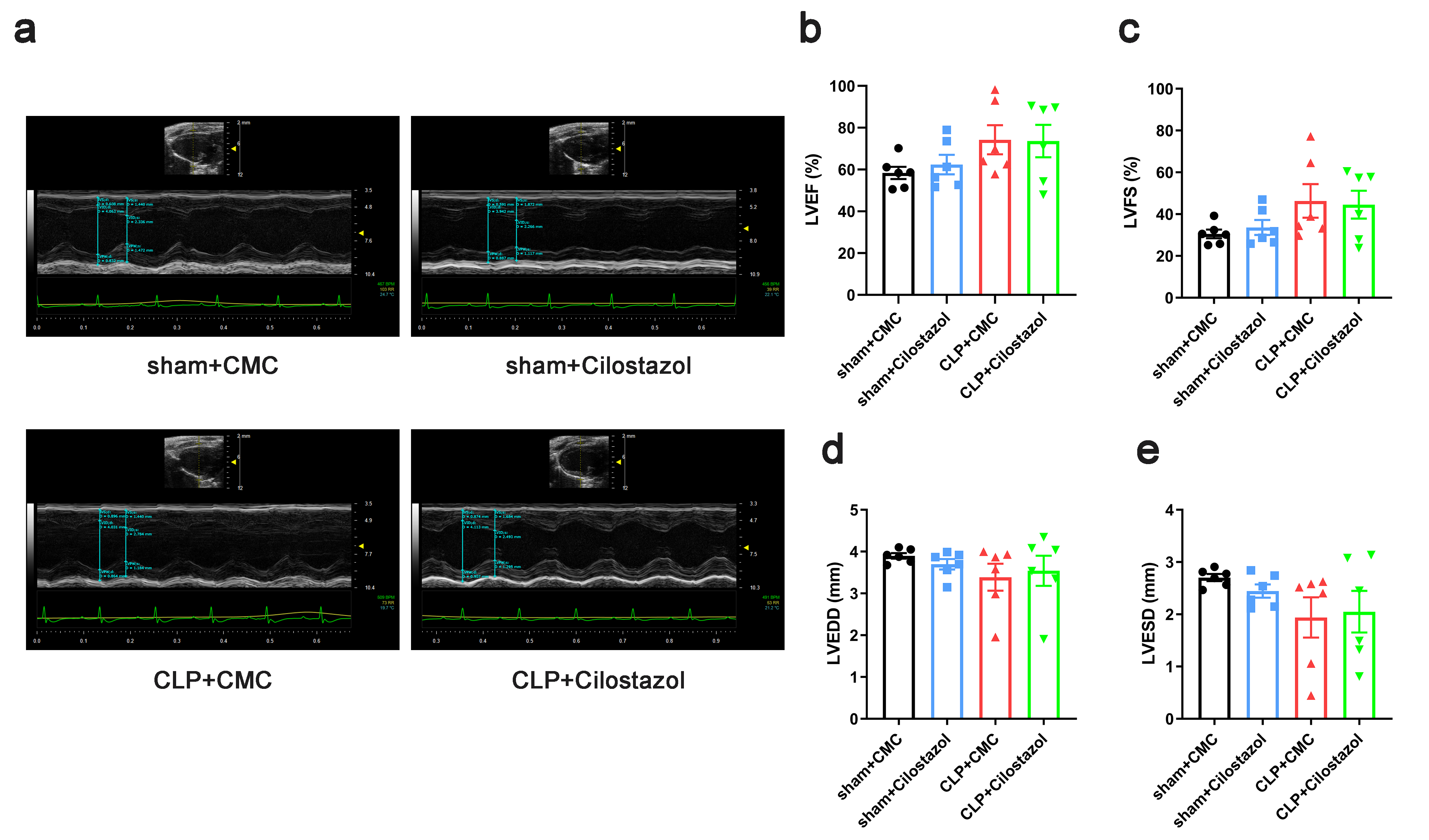

Supplement: Supplementary file 5 — Additional file 5: Fig. S5. Effect of cilostazol treatment on cardiac function after CLP. a Representative M-mode images of the four different groups. b-e Quantitative analysis of LVEF (b), LVFS (c), LVEDD (d), and LVESD (e) in each group (n = 6). These data indicate no significant difference. [file 10020_2022_556_MOESM5_ESM.tif]

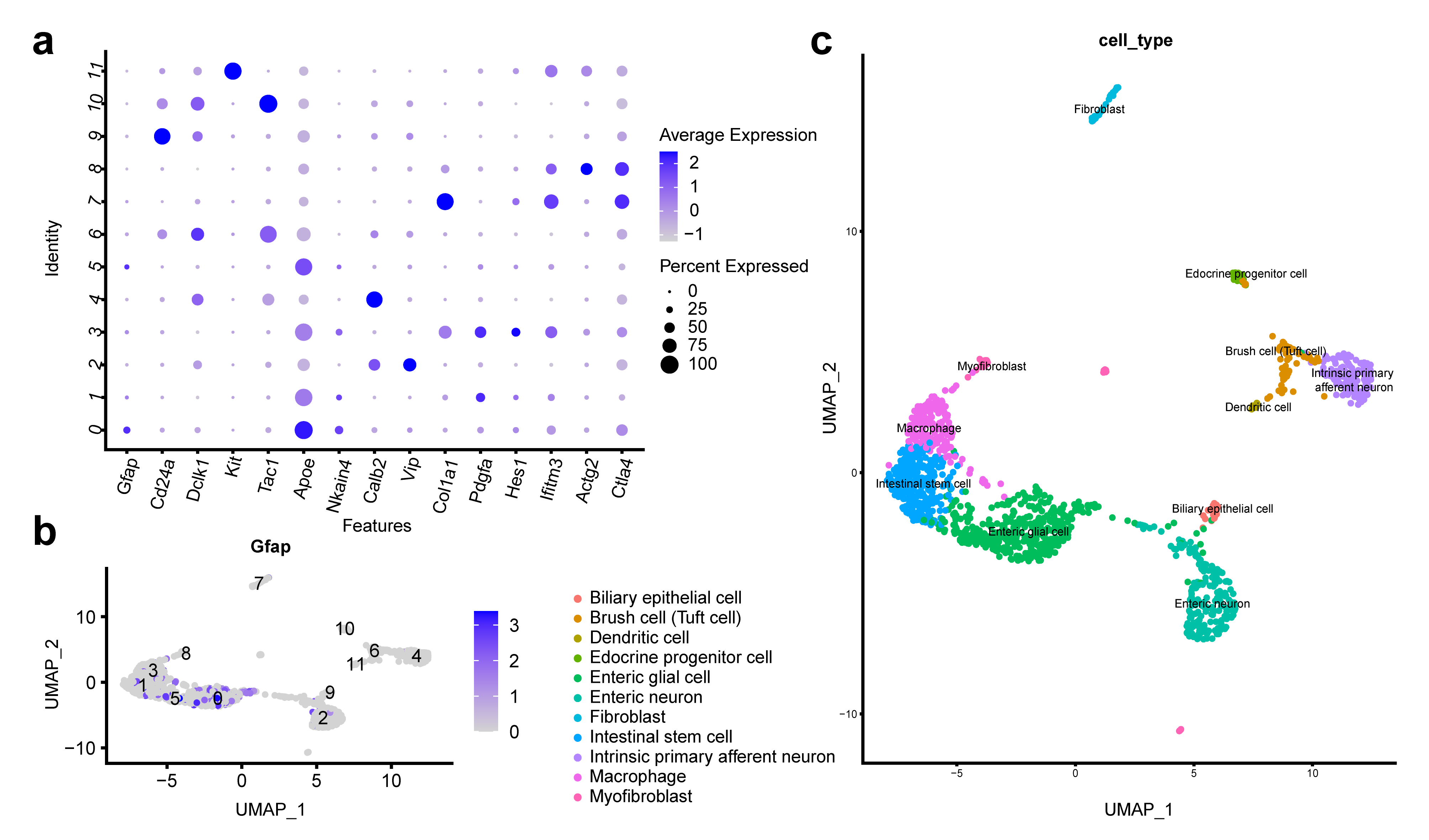

Supplement: Supplementary file 6 — Additional file 6: Fig. S6. Single-cell sequencing analysis of adult mice intestine. a Dotplot for highest specificity gene markers of cell clusters. Dot size represents the percentage of cells expressing the denoted gene, and the color represents average normalized expression level within the denoted cluster. b The expression of GFAP in different cell clusters by analysis of the Uniform Manifold Approximation and Projection (UMAP). c Different cell subpopulation of the GSE156905 dataset derived from the adult mice intestine. [file 10020_2022_556_MOESM6_ESM.tif]

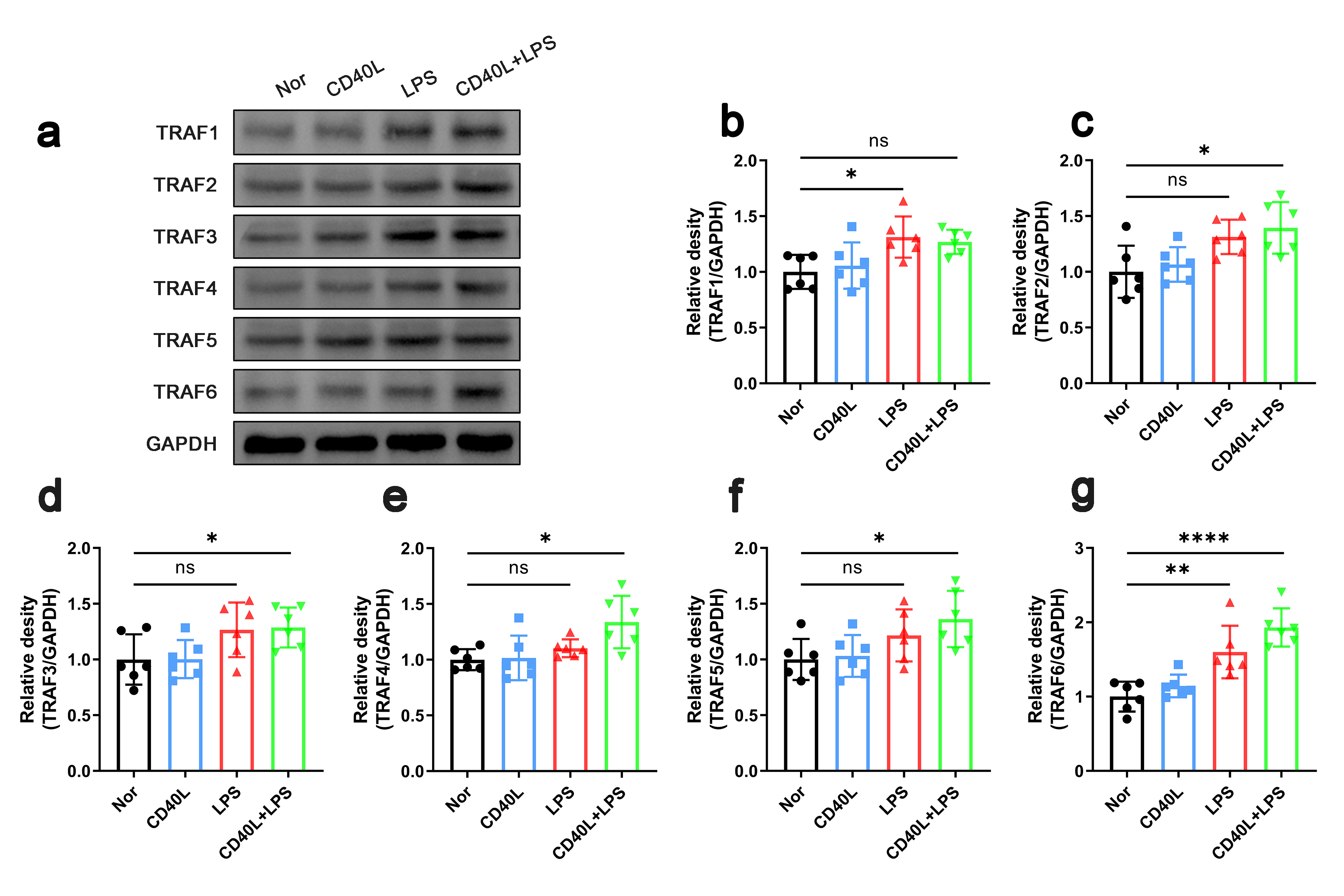

Supplement: Supplementary file 7 — Additional file 7: Fig. S7. Effect of LPS and CD40L on TRAFs proteins in EGCs. a Representative western blot images of TRAFs proteins expression in EGCs. b-g Western blot analysis of TRAF1 (b), TRAF2 (c), TRAF3 (d), TRAF4 (e), TRAF5 (f) and TRAF6 (g) expression in different groups of EGCs (n = 6). The data are presented as the mean ± SEM, *P < 0.05, **P < 0.01, ***P < 0.001, ****P < 0.0001. [file 10020_2022_556_MOESM7_ESM.tif]
